# Together Against COVID-19 Concerns: The Role of the Dyadic Coping Process for Partners’ Psychological Well-Being During the Pandemic

**DOI:** 10.3389/fpsyg.2020.578395

**Published:** 2021-01-07

**Authors:** Silvia Donato, Miriam Parise, Ariela Francesca Pagani, Margherita Lanz, Camillo Regalia, Rosa Rosnati, Raffaella Iafrate

**Affiliations:** Department of Psychology, Family Studies and Research University Centre, Universitá Cattolica del Sacro Cuore, Milan, Italy

**Keywords:** COVID-19, couple relationship, dyadic coping, stress communication, satisfied, dissatisfied couples

## Abstract

The situation caused by the 2019 coronavirus disease (COVID-19) has been representing a great source of concern and a challenge to the psychological well-being of many individuals around the world. For couples in particular, this extraordinary rise in concern, combined with the stress posed by the virus containment measures, such as prolonged cohabitation and lack of support networks, may have increased the likelihood of couple problems. At the same time, however, COVID-19 concerns may have been a stimulus to activate couples’ stress management processes. A couple’s resource, which may have an important role in dealing with COVID-19 concerns and stress, is dyadic coping, i.e., the process through which partners face stress together. Drawing on a sample of 1,823 Italian individuals involved in a couple relationship, the current study tested a serial mediation model in which concerns about COVID-19 predicted psychological well-being, through both explicit stress communication and perceived partner dyadic coping responses. In addition, the study explored whether this dyadic coping process functioned the same way in satisfied and dissatisfied couples. Results showed that concerns about the situation related to COVID-19 significantly threatened individuals’ psychological well-being. However, these concerns positively predicted explicit stress communication, which in turn positively predicted perceived partner’s dyadic coping responses, which finally positively predicted psychological well-being. In addition, in the group of dissatisfied individuals, the association between explicit stress communication and perceived partners’ dyadic coping responses was not significant. The present study adds to the research on couples’ coping by testing for the first time the whole theoretical model of dyadic coping and does so during a global emergency situation. The study also suggests key components of preventive interventions for individuals in couples.

## Introduction

After its identification in China at the end of 2019, a novel coronavirus, the severe acute respiratory syndrome coronavirus 2 (SARS-CoV-2), has spread worldwide, causing a pandemic of respiratory illness called 2019 coronavirus disease (COVID-19). COVID-19 has been representing a major threat to global human health (World Health Organization [WHO], 2020). COVID-19, in fact, can be extremely severe and, in some cases, can cause death, especially in the elderly and in people already affected by other diseases (Jordan et al., 2020). COVID-19 has also rapidly emerged as a threat to global economy (World Trade Organization [WTO], 2020). In fact, the lockdown and shutdown policies have led to economic difficulties, and many people and their families are experiencing job instability or loss, financial hardship, and, in general, uncertainty for the economic future (United Nations, 2020). Moreover, the situation related to COVID-19 has been posing a threat to social and interpersonal relationships (Brooks et al., 2020; Carlson et al., 2020). Especially during the phase of strict lockdown, people were mandated to self-isolate at home–and work at home when possible–and movement was strictly restricted. This resulted, on the one hand, into a forced and prolonged cohabitation with one’s immediate family, such as the partner or children, and, on the other hand, into a limited possibility of physical proximity with one’s not cohabiting family members, friends, and community. Nowadays, the evolution of the situation is still uncertain, and second waves of the pandemic during Fall 2020 have already required new lockdown measures in Italy and around the world. Taken together, all these aspects represent a great source of stress, concerns, and fear, which challenge the mental health and well-being of many individuals around the world (Brooks et al., 2020; Satici et al., 2020). For couples in particular, this extraordinary rise in stress, together with the combination of confinement and isolation, may have increased the likelihood of couple’s problems, as indicated by the significant upsurge in divorce applications in China in March 2020 (Deese, 2020; Global Times, 2020).

According to the Vulnerability Stress and Adaptation (VSA) model (Karney and Bradbury, 1995), managing common stressors is one of the major tasks couple members are required to complete while navigating their daily life: Stress related to the COVID-19 situation could therefore activate partners’ stress management processes. According to Bodenmann’s Systemic-Transactional Model of dyadic coping Bodenmann (1995, 1997, 2005), when partners deal with a stressor affecting them both directly and simultaneously, such as in the COVID-19 emergency, the source of stress is defined as common, and dyadic stress is observed. To cope against dyadic stress, partners can initiate a dyadic coping process, which is the interplay between both partners’ stress and coping reactions as well as proper common responses to the dyadic stressor. Both experimental and correlational studies, in fact, showed that, when facing dyadic stressors, partners engage in dyadic coping to recover from the stressful situation (Meuwly et al., 2012; Bertoni et al., 2015). More specifically, the dyadic coping process is depicted as a cycle in which the experience of stress becomes a dyadic issue when partners communicate about it. Stress communication is therefore the first step in the dyadic coping process. Research has shown that couples in which both partners communicate openly reported higher levels of satisfaction than those who communicate without examining the events or their moods (Christensen and Shenk, 1991; Guerrero et al., 2011). Explicit stress communication is then important to avoid misunderstanding and to elicit congruent dyadic coping responses (Kuhn et al., 2017). Once stress is communicated, in fact, one partner’s communication is appraised, decoded, and evaluated by the other partner, who then reacts with his/her coping responses. Partners’ coping responses can be positive as well as negative: positive dyadic coping occurs when one partner responds supportively to the other’s stress signals, showing understanding and being helpful, or when both partners engage in a joint management of the stressor. Negative dyadic coping occurs when one partner responds with disinterest, sarcasm, or belittlement to the other’s stress signals. In general, dyadic coping is, firstly, aimed at restoring or maintaining both partners’ psychological well-being, by reducing the partners’ levels of stress, and, secondly, at enhancing couple functioning, by strengthening partners’ sense of we-ness and reciprocal trust.

In addition to research demonstrating the role of dyadic coping for partners’ relational well-being (e.g., Donato et al., 2015; Hilpert et al., 2016; Parise et al., 2019), abundant research has proven that coping positively as a couple in times of stress significantly reduces partners’ distress and improves partners’ psychological health, both when dealing with normative (Molgora et al., 2018, 2019; Alves et al., 2019) and non-normative life events (Badr et al., 2010; Meier et al., 2011; Rottmann et al., 2015).

The COVID-19 emergency is a non-normative life event of a particular intensity and extraordinary nature in which dyadic coping could play an important role in maintaining partners’ psychological well-being despite the numerous sources of stress, concerns, and fear characterizing the situation connected to the epidemic and lockdown restrictions. A recent study, in fact, showed that how the partner responds to the other’s COVID-19-related stressors protect individuals from the negative effects of COVID-19-related stressors (Balzarini et al., 2020).

### The Current Study

The current study was aimed at investigating whether and how the concerns related to the COVID-19 situation activated partners’ dyadic coping process, and whether this, in turn, contributed to partners’ psychological well-being. In particular, on the basis of the Systemic-Transactional Model of dyadic coping (Bodenmann, 1995, 1997, 2005) and on the empirical research reviewed above, we intended to test a serial mediation model, in which concerns about COVID-19 predicted explicit stress communication, which in turn predicted perceived partner dyadic coping responses, which finally predicted psychological well-being.

In addition, a secondary objective of the present study was to exploratorily examine whether this dyadic coping process functions the same way in satisfied and dissatisfied couples, presuming that dissatisfied couples may present a less effective and functional dyadic coping process.

With regard to the first objective, we expected COVID-19 concerns to be negatively associated with psychological well-being (H1), as the literature has widely shown that stressors negatively affect psychological well-being (Thoits, 2010; Schönfeld et al., 2016). Moreover, although the dyadic coping model assumes that partners’ concerns for the stressful situation trigger one’s stress communication to the other partner, no studies to date tested this specific association. We expected that stress was also positively associated with stress communication in the COVID-19 emergency (H2), as the partner is regarded as the most important source of support in times of stress, that is not easily substituted (Coyne and De Longis, 1986; Dakof and Taylor, 1990; DeLongis et al., 2010). In addition, literature has started to show that stress communication is linked to partners’ dyadic coping responses (Kuhn et al., 2017), especially when it is explicit. Explicit stress communication, in fact, was found to be associated with one partner’s perceptions of the other’s responsive dyadic coping (Pagani et al., 2019). We therefore expected stress communication to positively predict the partner’s dyadic coping responses in the context of the COVID-19 emergency as well (H3). Finally, since dyadic coping responses were found to be associated with psychological well-being (e.g., Bodenmann et al., 2011; Rusu et al., 2015), we expected this association to be significant and positive also in the COVID-19 emergency (H4).

With regard to our secondary objective, we expected that in dissatisfied couples, the process of dyadic coping could be somehow disrupted, since research has shown that distressed partners differ from partners who are not in distress in the way in which they exchange support and interact with each other (Whisman et al., 2008; Verhofstadt et al., 2013). Hypotheses 2, 3, and 4 above could be further specified as a function of the potential moderating role of relationship satisfaction. In particular, the association between COVID-19 concerns and explicit stress communication could not be significant for dissatisfied couples who may seek support outside the couple itself (H2a). Moreover, in dissatisfied couples, even when communicated explicitly, stress communication could not activate a dyadic coping response from the partner (H3a). Indeed, dissatisfied partners’ communication, although explicit and direct, may be subtly connoted by blame and criticism, thereby discouraging partner supportive responses. Research has found in fact that distressed couples show less positive and more negative support-seeking strategies than non-distressed ones (Verhofstadt et al., 2013). Alternatively, despite explicit stress communication, a dissatisfied partner may not be willing to offer support. Finally, we expected a non-significant or negative association between partner dyadic coping responses and psychological well-being (H4a). Dissatisfied partners, in fact, might be less skillful or effective in enacting dyadic coping responses. Dissatisfied couples were found to be characterized by less positive (e.g., trust, support) and more negative dimensions (e.g., emotional distance, disengagement) as well as less cooperative conflict styles than satisfied couples (Bertoni and Bodenmann, 2010). It is also possible that a dissatisfied partner could interpret the other dyadic coping as less responsive to his/her needs, as dissatisfied partners were found to be less benevolent when interpreting the other’s behaviors (Bradbury and Fincham, 1990).

The investigation of the role of dyadic coping is particularly relevant for both research and intervention. To the best of our knowledge, this is the first study to test the complete process of dyadic coping in one model and, in particular, to test this model applied to the specific COVID-19 situation. Moreover, this study may help practitioners to identify the resources to enhance and protect partners’ well-being. The identification of the resources aimed at maintaining mental well-being of individuals, and especially those in vulnerable groups, has in fact been defined as a priority during this epidemic and is important for the implementation of preventive interventions tailored on individuals’ specific needs in the current and future emergency situations (Holmes et al., 2020).

## Materials and Methods

### Participants and Procedure

The present study is part of a broader research project, titled “The Family at the time of COVID-19,” developed by the Family Studies and Research University Centre of the Università Cattolica del Sacro Cuore (Milan, Italy) and conducted in collaboration with the Human Highway Society. This research originally included a representative sample of the Italian population (*N* = 2,999), but for the purpose of the present study, we selected people reporting to be in a couple relationship (*N* = 1,823). In this sub-sample, women were 67.4% (*N* = 1,228) and men 32.6% (*N* = 595). In terms of age, 0.7% of participants were between 18 and 24 years old, 13.9% between 24 and 34 years old, 34.1% between 35 and 44 years old, 32.1% between 45 and 54 years old, 14.6% between 55 and 64 years old, and 4.6% were over 65 years old. Overall, 71.6% of participants were married, whereas 28.4% were cohabiting without being married. Moreover, 73.5% of participants were parents, whereas 26.5% had no children.

The data were collected from March 30th to April 7th, during the Italian lockdown phase (started on the 11th of March), with a self-report questionnaire disseminated through different platforms and mainstream social media. A brief presentation informed the participants about the aims of the study, and an electronic informed consent was requested from each participant before starting the investigation. To guarantee anonymity, no personal data, which could allow the identification of participants, were collected. Due to the aim of the current research, the only inclusion criterion was to be over 18 years old. The study was approved by the Ethics Committee of the Department of Psychology of the Università Cattolica del Sacro Cuore (protocol number 15–20).

### Measures

#### COVID-19 Concerns

Participants expressed their degree of concern about the situation related to COVID-19 with the item “To what extent are you concerned about the current COVID-19-linked situation?” They were asked to respond on a 7-point Likert scale, from 1 = *not at all* to 7 = *extremely*.

#### Explicit Stress Communication

Participants indicated the degree to which they communicated explicitly their level of stress related to the COVID-19 situation to their partner with the item “To what extent did you communicate explicitly your stress related to the current COVID-19-linked situation to your partner?” Participants responded on a 5-point Likert scale, from 1 = *not at all* to 5 = *very much*.

#### Dyadic Coping

To assess dyadic coping responses, we used a shorter 8-item version of the original 41 items of the Dyadic Coping Questionnaire (DCI; Bodenmann, 1997; Donato et al., 2009). Participants were asked to assess their perceptions of the partner’s positive and negative dyadic coping responses (e.g., “My partner proposed practical solutions to the problems that this situation caused”; “My partner accused me of not managing stress well enough”) on a 5-point Likert scale ranging from 1 = *never* to 5 = *very often*. In this study, we averaged the 8 items of the scale to create a total index in which a higher score indicated a greater level of dyadic coping. The Cronbach’s alpha was 0.88.

#### Psychological Well-Being

Psychological well-being was measured through 4 items selected from the Mental Component Summary of the Short-Form Health Survey (SF-12; Apolone and Mosconi, 1998; Apolone et al., 2001). These items measure the overall participant’s psychological well-being in terms of vitality (having a lot of energy), mental health (feel calm and peaceful), and social functioning (interference of physical health or emotional problems with social activities). An item example is “I felt full of energy.” Participants were asked to report about their well-being over the previous week on a 6-point Likert scale ranging from 1 = *never* to 6 = *always*. We averaged the 4 items to create a total score in which a higher score indicated a greater level of psychological well-being. The Cronbach’s alpha was 0.71.

#### Couple Satisfaction

Couple satisfaction was measured through one *ad hoc* item. This item (“Overall, how do you rate the relationship with your partner during this period?”), measuring global perception of couple relationship satisfaction, was administered on a 10-point Likert scale (1 = *very negative* and 10 = *very positive*). On the basis of the theoretical range of the scale, dissatisfied individuals were operationalized as those scoring 5 or lower on this item, whereas satisfied ones as those scoring 6 or higher.

### Data Analyses

To test our hypotheses, we modeled the association between our predictor (i.e., COVID-19 concerns) and outcome (i.e., psychological well-being; H1). Moreover, we modeled the association between COVID-19 concerns and our first mediator (i.e., explicit stress communication; H2), the association between explicit stress communication and our second mediator (i.e., dyadic coping responses; H3), and finally the association between dyadic coping responses and psychological well-being (H4). In particular, we ran a serial mediation model using AMOS version 21 (Arbuckle, 2012). In this model, explicit stress communication and dyadic coping responses were treated as serial mediators of the association between concerns about the situation in relation to COVID-19 and psychological well-being. In line with the theoretical model, we tested the overall indirect effect of the two mediators together in the link between COVID-19 concerns and psychological well-being (i.e., from COVID-19 concerns to explicit stress communication to dyadic coping responses to psychological well-being) through the “SerialMediation” user-defined estimand provided by Gaskin (2016).

With regard to our secondary objective, a multi-group approach was used to test any differences in the hypothesized specific paths between the group of dissatisfied individuals (*N* = 165) and the group of satisfied individuals (*N* = 1,658). In particular, we tested the differences between the two groups in the association between COVID-19 concerns and explicit stress communication (H2a), between explicit stress communication and dyadic coping responses (H3a), and between dyadic coping responses and psychological well-being (H4a). For each specific path, the differences were examined by comparing a model in which all structural paths were allowed to vary across the groups with a model in which the target structural path was constrained to be equal between the groups. The Δχ^2^ was used to compare the models. In case the [Δχ^2^] was not significant, we retained more parsimonious, constrained model.

## Results

### Preliminary Analyses

Preliminary analyses showed that participants reported high levels of COVID-19 concerns (*M* = 6.18, *SD* = 0.97; range 3–7) and moderate levels of explicit stress communication (*M* = 3.73, *SD* = 0.91; range 2–5), dyadic coping responses (*M* = 3.58, *SD* = 0.72; range 1.38–5), and psychological well-being (*M* = 3.56, *SD* = 0.84; range 1–6). Moreover, COVID-19 concerns were negatively correlated with psychological well-being, but positively correlated with explicit stress communication and dyadic coping responses. Moreover, explicit stress communication was positively correlated with dyadic coping responses, but negatively correlated with psychological well-being. Finally, dyadic coping responses were positively correlated with psychological well-being (Table 1).

**TABLE 1:** Correlations, means, and standard deviations for the study variables.

Variables	1	2	3	4	*M* (Sat./Dissat.)	*SD* (Sat./Dissat.)
1. COVID-19 concerns (range 1–7)	–				6.18 (6.19/6.12)	0.97 (0.96/1.02)
2. Explicit stress communication (range 1–5)	0.20***	–			3.73 (3.76/3.36)	0.91 (0.89/1.03)
3. Dyadic coping responses (range 1–5)	0.05*	0.26***	–		3.58 (3.68/2.65)	0.72 (0.65/0.62)
4. Psychological well-being (range 1–6)	−0.27***	−0.18**	0.26***	–	3.56 (3.60/3.11)	0.84 (0.82/0.83)

N = 1683. * p < 0.05, ** p < 0.01, *** p ≤ 0.001. *Sat*. = satisfied individuals; *Dissat.* = dissatisfied individuals.

Moreover, we tested the differences between satisfied and dissatisfied individuals in the level of the variables. Satisfied and dissatisfied partners showed similar levels on COVID-19 concerns [*F*(1, 1,822) = 0.77, *p* = 0.379; dissatisfied individuals: *M* = 6.12, *SD* = 1.02, satisfied individuals: *M* = 6.19, *SD* = 0.96]. Nonetheless, in comparison with satisfied individuals, dissatisfied ones showed less explicit stress communication [*F*(1, 1,822) = 29.28, *p* = 0.000; dissatisfied individuals: *M* = 3.36, *SD* = 1.03, satisfied individuals: *M* = 3.76, *SD* = 0.89], less positive dyadic coping responses [*F*(1, 1,822) = 159.06, *p* = 0.000; dissatisfied individuals: *M* = 2.65, *SD* = 0.62, satisfied individuals: *M* = 3.68, *SD* = 0.65], and lower psychological well-being [*F*(1, 1,822) = 36.43, *p* = 0.000; dissatisfied individuals: *M* = 3.11, *SD* = 0.83, satisfied individuals: *M* = 3.60, *SD* = 0.82].

### Testing the Serial Mediation Model

**FIGURE 1:**
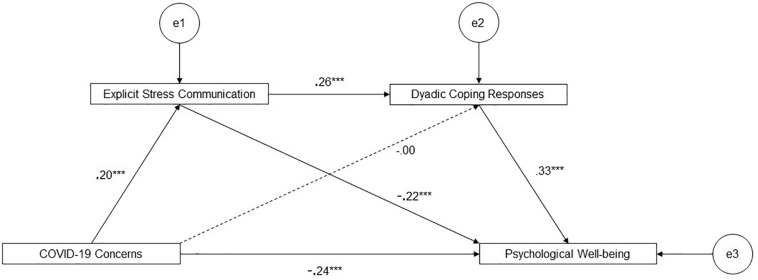
Serial mediation model. Path coefficients are standardized estimates. ***p ≤ 0.001.

With regard our first objective, the serial mediation model explained overall the 19% of variance of psychological well-being. As Shown in Figure 1, concerns about the situation related to COVID-19 negatively predicted psychological well-being (β = −0.24, *p* = 0.001; H1). Moreover, COVID-19 concerns positively predicted explicit stress communication (β = 0.20, *p* = 0.001; H2), which in turn positively predicted perceived partner’s dyadic coping responses (β = 0.26, *p* = 0.001; H3), which in turn positively predicted psychological well-being (β = 0.33, *p* = 0.001; H4). Testing the significance of the overall indirect effect revealed that, as hypothesized, explicit stress communication and dyadic coping responses serially mediated the link between concern about the situation related to COVID-19 (β = 0.02, *p* = 0.001, 95% CI = 0.01, 0.02) and psychological well-being. This indirect pathway partially accounted for the overall impact of concerns on psychological well-being, given that the direct effect remained significant (β = −0.24, *p* = 0.001)^1^. Although not a primary focus of the current study, another effect was found to be significant: explicit stress communication negatively predicted participants’ psychological well-being.

**FIGURE 2:**
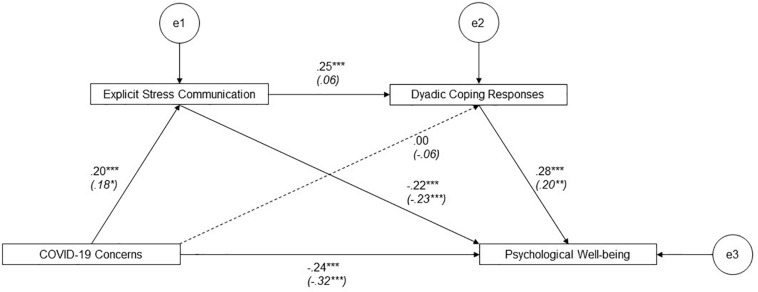
Serial mediation model for satisfied *vs*. dissatisfied partners. Path coefficients are standardized estimates. Dissatisfied partners’ coefficients appear in brackets. *p = 0.05; **p < 0.01; ***p ≤ 0.001.

As for our second objective, as shown in Figure 2, the multi-group analyses showed that the association between explicit stress communication and perceived partner’s dyadic coping responses was significantly different for the two groups (Δχ^2^ = 7.42, *p* = 0.006; H3a). Specifically, in the group of satisfied individuals, this association was positive and significant (β = 0.25, *p* = 0.001), whereas in the group of dissatisfied individuals, no association was found (β = 0.07, *p* = 0.409). No differences between the groups were found with regard to the other specific pathways tested (i.e., COVID-19 concerns → explicit stress communication, H2a; dyadic coping responses → psychological well-being, H4a).

## Discussion

The present study was intended to examine whether and how the concerns related to the COVID-19 situation activated partners’ dyadic coping process, and whether this process predicted partners’ psychological well-being. In particular, we tested a serial mediation model in which concerns about COVID-19 predicted psychological well-being, through both explicit stress communication and perceived partner dyadic coping responses. Moreover, we also explored whether the above dyadic coping process in response to COVID-19 concerns was similar or different in satisfied and dissatisfied partners.

Results of preliminary analyses showed that in general, participants were very worried about the situation related to COVID-19, while showed adequate levels of explicit stress communication, dyadic coping, and psychological well-being. This is not surprising as our research design aimed to collect data from a community, rather than a clinical sample, and showed how the COVID-19 worries were common and widespread. Nonetheless, in this community sample, dissatisfied partners appeared in this situation as more vulnerable than satisfied ones. Despite similar levels on COVID-19 concerns, in fact, dissatisfied partners showed less explicit stress communication, less positive dyadic coping responses, and lower psychological well-being than satisfied partners. These results were in line with our hypotheses that dissatisfied partners are more at risk than satisfied ones, in that they present lower levels of relational resources and individual well-being. In addition, our findings further highlight how dissatisfied partners present not only fewer but also less effective resources, as shown by the analyses related to our second objective.

In line with the pattern emerged from the intercorrelations among variables, findings of the model revealed that in general, the concerns about the situation related to COVID-19 positively predicted explicit stress communication, which in turn positively predicted perceived partner’s dyadic coping responses, which finally positively predicted psychological well-being. Once accounted for the effects of mediators, the link between concerns about the COVID-19 situation and psychological well-being remained significant, thereby showing partial mediation. These results highlight how COVID-19 concerns significantly threaten individuals’ psychological well-being, in terms of energy, mental health, and social functioning, confirming our first hypothesis (H1). The COVID-19 situation may have induced intense feelings of concern, due to the seriousness of the health emergency, the consequent economic crisis, job instability, and uncertainty about the future (Ferrucci et al., 2020; Mazza et al., 2020; Pagnini et al., 2020; Rossi et al., 2020), also in couples (Günther-Bel et al., 2020; Panzeri et al., 2020; Rapelli et al., 2020). These concerns, likely amplified by stressors related to the virus containment measures (e.g., prolonged co-habitation, lack of formal and informal support networks, etc.), may have hampered people’s personal well-being. Although stressors, such as the current pandemic, are not necessarily avoidable, nor are fears and concerns related to them (which have vital functions for the individual; e.g., Mobbs et al., 2015), our study shows that we can draw on couples’ resources to effectively deal with them.

According to the present research, one of these resources for couples is dyadic coping. It has been demonstrated, in fact, that appealing to fear as a measure of behavioral change can be effective during a global stressor, such as a pandemic, only when people possess (or are helped to acquire) a sense of efficacy to deal with the threat (Witte and Allen, 2000). Research already highlighted that individual coping resources are key factors promoting adjustment to the COVID-19 emergency (Vagni et al., 2020a,b). Our study underlines that also promoting couples’ dyadic coping competences can be a way to enhance partners’ ability to deal with the stress and concerns related to the epidemic (Prime et al., 2020) and adds to the literature showing that pro-relationship processes in response to negative events are important for couples (see Donato and Parise, 2015). In particular, in line with our second hypothesis (H2), our model showed that higher concerns about the COVID-19 situation predicted a more explicit communication of one’s stress to the partner, which is the first step of the dyadic coping process. Being the partner the most important source of support for individuals in a couple relationship, our study showed that COVID-19 concerns can also be a stimulus to activate a couple’s resource through the stress communication. To our knowledge, this is the first study that tested the specific assumption of the dyadic coping model that refers to the connection between stress and stress communication.

Explicit stress communication, on the other hand, was positively associated with perceived partners’ dyadic coping responses, confirming our third hypothesis (H3). This finding is in line with the recent literature focusing on the role of stress communication in the dyadic coping process and specifically with the evidence that explicit stress communication is associated with one partner’s perceptions of the other’s responsive dyadic coping (Pagani et al., 2019). Explicit stress communication may help partners to avoid misunderstandings and to attune with the partner’s support needs.

Finally, in accordance with our fourth hypothesis (H4), perceived partner dyadic coping responses were found to be positively associated with psychological well-being. As already shown by the literature, dyadic coping plays a critical role in stress reduction and in restoring well-being after a stressful experience (Bodenmann et al., 2011; Rusu et al., 2015). In particular, our study points to the importance of dyadic coping for psychological well-being during the COVID-19 emergency.

Although not a primary focus of the current study, we also found evidence for another effect: explicit stress communication negatively predicted participants’ psychological well-being. It is possible that more explicit stress communication is a marker of participants’ higher distress in front of the COVID-19 emergency. In line with a previous study, in fact, interpersonal communication with significant others about COVID-19 was associated with greater perceived stress (First et al., 2020).

An additional aim of our study was to test whether the dyadic coping process in response to COVID-19 concerns was similar or different in satisfied and dissatisfied partners, hypothesizing that dissatisfied ones could present a less effective and functional dyadic coping. Dissatisfied individuals showed on average lower levels of explicit stress communication and dyadic coping responses. The associations between COVID-19 concerns and explicit stress communication and between perceived partners’ dyadic coping responses and psychological well-being were significant and similar for both satisfied and dissatisfied individuals, thereby not confirming our hypotheses H2a and H4a. Nonetheless, the association between explicit stress communication and perceived partners’ dyadic coping responses was not significant in dissatisfied individuals, in line with our hypothesis H3a.

It appears that the weak link in the dyadic coping process for dissatisfied couples is the pathway from the explicit stress communication to the partner’s dyadic coping responses. This is maybe due to a lack of competence in stress communication by the stressed partner (Verhofstadt et al., 2013), to a lower proneness to respond supportively to the other’s stress communication (Bertoni and Bodenmann, 2010), or to a lower ability to detect the partner’s dyadic coping responses (Bradbury and Fincham, 1990). These could be specific liabilities of dissatisfied couples in dyadic coping. In line with the VSA model (Karney and Bradbury, 1995), pre-existing vulnerabilities may interfere with dyadic adaptation processes, such as dyadic coping, and may exacerbate the effects of pandemic-related stressors (Pietromonaco and Overall, 2020; Prime et al., 2020). Similarities with satisfied couples on the other components of the model, however, reveal that the dyadic coping process, once these liabilities are addressed, could be an important resource for dissatisfied individuals as well.

This finding, however, needs to be confirmed by further research specifically designed to test this comparison, since a limitation of our study is that only a small sub-group of dissatisfied individuals was collected. Moreover, only one partner of the couple was involved in the research, thereby preventing us from detecting the interplay between the couple’s members. As another limitation, this study was correlational; therefore, the associations found cannot be interpreted in causal terms. Future longitudinal research may help empirically establish the direction of effects. Third, a single *ad hoc* item was used to measure COVID-19 concerns, explicit stress communication, and couple satisfaction. In particular, a single-item measure of explicit stress communication may have limited us in capturing the complexity and quality of individuals’ stress communication process. Future research using multidimensional measures of stress communication may help to better understand the link between stress communication and dyadic coping responses, especially in dissatisfied individuals. Finally, we did not measure how partners respond not only to stressors and concerns but also to positive events during the emergency, as responses to positive events (i.e., capitalization) are an important form of coping (Langston, 1994; Pagani et al., 2020) that research found to be linked with dyadic coping responses (Donato et al., 2018). Future research should be devoted to test this association.

Notwithstanding the above limitations, the present results highlight the importance of the dyadic coping process as a protective response to COVID-19 concerns and call for a more attentive examination of the communication component of the dyadic coping process, especially in dissatisfied couples. More specifically, the present results point to the following implications for intervention: each step of the process we tested can be a useful target for intervention aimed at preventing the negative impact of the COVID-19 situation (or future emergencies) on individuals in couples. Preventive efforts should be devoted to help partners mitigate their concerns by promoting an optimistic outlook on the stressful situation, which was found to be crucial for couples’ functioning in front of potentially distressful situations (Parise et al., 2017). Secondly, interventions could be aimed at improving partners’ stress communication strategies in order to make it more explicit. Explicit communication helps avoid misunderstandings and provide a more responsive support (Pagani et al., 2015, 2019). Finally, given the role of dyadic coping responses in the promotion of psychological well-being and relationship quality (e.g., Donato and Parise, 2012; Donato et al., 2014; Canzi et al., 2019), efforts should be directed to improve partners’ dyadic coping competences. Training on key interpersonal competences is in fact an important component of preventive interventions for families (e.g., Ledermann et al., 2007; Bertoni et al., 2017).

## Data Availability Statement

The raw data supporting the conclusions of this article will be made available by the authors, without undue reservation.

## Ethics Statement

The studies involving human participants were reviewed and approved by the Ethics Committee of the Department of Psychology of the Università Cattolica del Sacro Cuore (protocol number: 15-20). The patients/participants provided their written informed consent to participate in this study.

## Author Contributions

SD, MP, and AP contributed to the development of the theoretical framework and to the writing of the manuscript. MP performed statistical analyses. ML, CR, RR, and RI coordinated the project. All authors contributed to manuscript revision, read and approved the submitted version.

## Conflict of Interest

The authors declare that the research was conducted in the absence of any commercial or financial relationships that could be construed as a potential conflict of interest.

## Footnotes

1 Given that the Northern regions of Italy were more severely impacted by the epidemic, we added participants’ area of residence (Northern Italy vs. Rest of Italy) as a control variable in the model. Results did not change meaningfully.
